# Targeting the COP9 signalosome for cancer therapy

**DOI:** 10.20892/j.issn.2095-3941.2021.0605

**Published:** 2022-03-21

**Authors:** Wenqi Du, Ruicheng Zhang, Bilal Muhammad, Dongsheng Pei

**Affiliations:** 1 Department of Pathology, Xuzhou Medical University, Xuzhou 221004, China; 2 Department of Human Anatomy, Xuzhou Medical University, Xuzhou 221004, China; 3 Department of Neurology, Affiliated Hospital of Xuzhou Medical University, Xuzhou 221006, China

**Keywords:** COP9 signalosome, ubiquitin, cullin-RING ligases, cell proliferation, tumorigenesis

## Abstract

The COP9 signalosome (CSN) is a highly conserved protein complex composed of 8 subunits (CSN1 to CSN8). The individual subunits of the CSN play essential roles in cell proliferation, tumorigenesis, cell cycle regulation, DNA damage repair, angiogenesis, and microenvironmental homeostasis. The CSN complex has an intrinsic metalloprotease that removes the ubiquitin-like activator NEDD8 from cullin-RING ligases (CRLs). Binding of neddylated CRLs to CSN is sensed by CSN4 and communicated to CSN5 with the assistance of CSN6, thus leading to the activation of deneddylase. Therefore, CSN is a crucial regulator at the intersection between neddylation and ubiquitination in cancer progression. Here, we summarize current understanding of the roles of individual CSN subunits in cancer progression. Furthermore, we explain how the CSN affects tumorigenesis through regulating transcription factors and the cell cycle. Finally, we discuss individual CSN subunits as potential therapeutic targets to provide new directions and strategies for cancer therapy.

## Introduction

The ubiquitin-proteasome system (UPS) detects post-translational modification of proteins, and controls a wide range of cellular processes including protein degradation, signal transduction, transcriptional regulation, DNA repair, and cell cycle progression^1^. The degradation of proteins by the UPS begins with the addition of multiple ubiquitin molecules to lysine residues in proteins^2^. The binding of ubiquitin to substrate proteins is mediated by a cascade of enzymatic reactions involving E1 ubiquitin-activating enzymes, E2 ubiquitin-conjugating enzymes, and E3 ubiquitin ligases^3,4^. E3, acting at the end of the three-enzyme cascade, controls eukaryotic biological processes by promoting protein ubiquitination and degradation^5^. Cullin-RING ligases (CRLs), a superfamily of E3 complexes, are organized on the basis of various cullin scaffold proteins (CUL1, CUL2, CUL3, CUL4A, CUL4B, and CUL5) and use interchangeable substrate receptors to recruit various substrates onto a common catalytic scaffold^6^. CRLs are involved in the regulation of various dynamic cellular processes critical for cancer cell survival^7^. Cullins are modified by NEDD8, a well-studied ubiquitin-like protein that critical in a variety of cellular processes. Neddylation is the process of NEDD8 binding to a substrate protein, thereby promoting the E3 activity of CRLs and modulating protein activity and function^8,9^. Previous studies have demonstrated that remodeling of CRLs is initiated by the cleavage of NEDD8 from CRLs, catalyzed by the deneddylase COP9 signalosome (CSN)^10,11^.

The CSN, originally identified as a repressor complex of light activated development in *Arabidopsis*, is a multifunctional protein complex comprising 8 subunits (CSN1–CSN8)^12^. The biological function of the CSN is often determined through the regulation of CRLs, which are involved in the regulation of numerous cellular processes^13^. In the past few years, our laboratory has published articles exploring the mechanisms underlying the roles of the CSN in human cancers. In this review, we aim to summarize current understanding and discoveries made by our laboratory and others regarding all subunits of the CSN in cancer initiation and progression. We first briefly describe the structural and functional features of the CSN, a key regulator at the intersection between neddylation and ubiquitination that is associated with tumor progression. We then discuss critical issues including the roles of individual CSN subunits in cancer progression. We explain how the CSN affects tumorigenesis through regulating transcription factor stability and transcriptional activity. CSN-mediated regulation of cell cycle progression is also involved in tumorigenesis. In addition, we review the essential signaling pathways associated with CSN subunits and the potential therapeutic targets in human cancer. Finally, we discuss the CSN’s emerging roles in carcinogenesis and highlight its promise as a potential target for cancer therapy.

## Architecture and functional features of the CSN

**Figure 1:**
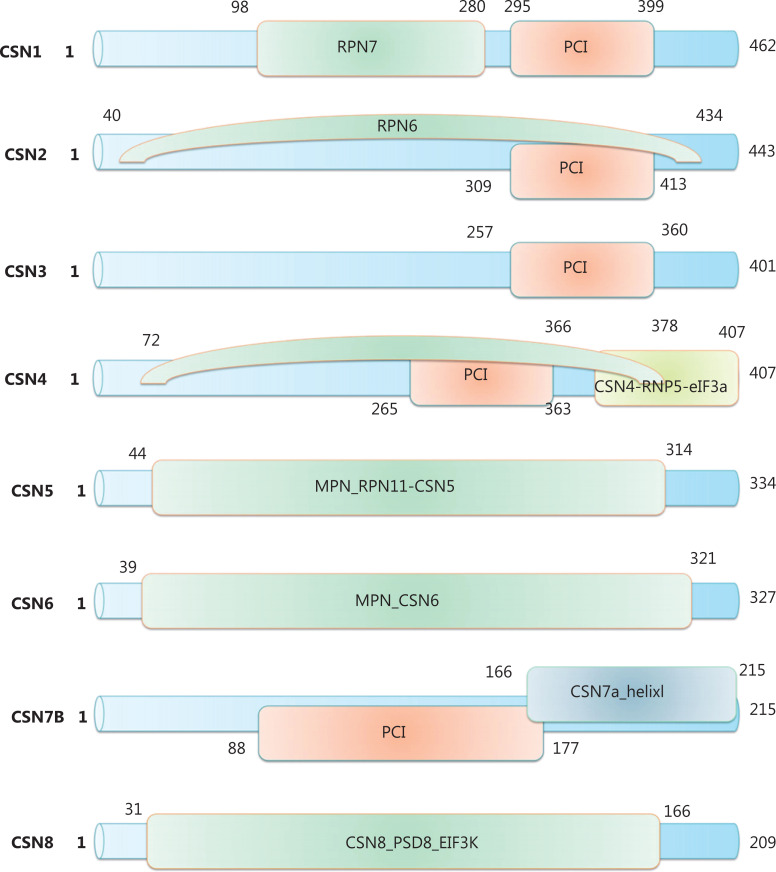
Schematic representation of the domain composition of CSN subunits.

COP9 is a multi-subunit protein complex that is a hallmark of eukaryotic cells and was first isolated from cauliflower^14,15^. The COP9 signalosome complex consists of 8 subunits (CSN1 to CSN8 from largest to smallest)^16^. Although CSN was initially identified as a repressor of photomorphogenesis in *Arabidopsis*^17,18^, the CSN subunit has been found to significantly affect several cellular functions, including angiogenesis, cell cycle control, DNA repair, maintenance of DNA fidelity, and microenvironmental homeostasis, all of which are essential in tumorigenesis^19,20^. Among the 8 CSN subunits, 6 CSN proteins (in the three-dimensional structure order of CSN7-CSN4-CSN2-CSN1-CSN3-CSN8) contain a proteasome lid-CSN-initiation factor 3 (PCI) domain characterized by helical repeats followed by a winged-helix subdomain^21^. The remaining subunits, CSN5, also known as Jun activation domain-binding protein 1 (Jab1/CSN5), and CSN6, are the only 2 Mpr1-Pad1-N-terminal (MPN) domain-containing subunits situated on top of the helical bundle formed by the C-terminal α-helices of each CSN subunit (**Figure 1**)^22^. Lingaraju et al.^23^ have presented the crystal structure of the CSN and described the molecular architecture of the complex in detail. The crystal structure of the CSN reveals that the PCI helical repeat domains are responsible for binding SCFS^KP2/CKS1^, whereas the helical bundle enables Jab1/CSN5 to sense the assembly state of the CSN. Jab1/CSN5 is a metalloprotease with a conserved Jab1 MPN domain metalloenzyme (JAMM) motif, and it requires a zinc ion as an activator. CSN6 is deficient in this JAMM motif and thus is identified as an MPN negative (MPN–) subunit, whereas Jab1/CSN5 is an MPN positive (MPN+) subunit^24,25^. As described in the introduction, the CSN regulates CRL activity by removing the covalently bound activator NEDD8. This deneddylation activity is performed by the JAMM/MPN+ domain of Jab1/CSN5, although this domain is inactive in isolation^24,26^. Binding of neddylated CRLs to CSN is sensed by CSN4 and communicated to CSN5 with the assistance of CSN6, thus resulting in the activation of deneddylase^23^. Furthermore, previous studies have indicated that the CSN complex is a critical platform enabling Jab1/CSN5 to function as a deubiquitinating enzyme (DUB). Moreover, the CSN controls the neddylation status of cells through destabilization of the associated deneddylation enzyme 1 (DEN1) and through its intrinsic DUB activity^27,28^. Therefore, the CSN is a key regulator at the intersection between neddylation and ubiquitination, which are associated with cancer progression. The following sections introduce all subunits of the CSN involved in tumor development to provide a foundation for cancer therapy.

## The diverse roles of CSN subunits in cancer

A growing body of evidence indicates that CSN subunits play critical roles in various human cancers. We used the GEPIA database (http://gepia.cancer-pku.cn/index.html) to systematically assess the differential expression of CSN subunits in various types of cancers. In statistical analysis of the transcriptomic data of various cancers, the database identified the CSN gene expression profiles across various tumor samples and matched normal tissues. Here, we present only the types of human cancers with discrepancies in CSN expression (**Supplementary Figure S1**). Among the CSN subunits, Jab1/CSN5 is the most studied subunit that acts as an oncogene. However, evidence has indicated the involvement of CSN4 and CSN7 in human cancer. Herein, we describe recent advances in understanding of the roles of individual CSN subunits in cancer progression (**Table 1**).

**Table 1:** Roles of CSN subunits in human cancers

CSN subunit	Cancer type	Role in cancer	Mechanism	Reference
CSN1	HCC	Oncogene	Upregulates cyclin A2 expression	^30^
	Penile squamous cell carcinoma	Tumor suppressor	CSN1 mutations result in aberrant miRNA processing	^31^
CSN2	HCC	Oncogene	CSN2 loss decreases radiation induced cell migration and EMT	^40^
	Breast cancer	Oncogene	Blocks ubiquitination and degradation of Snail	^39^
	CRC	Tumor suppressor	CSN2 is a potential target of miR-15a-3p	^36^
	Gastric cancer	Tumor suppressor	Low serum CSN2 indicates unfavorable survival	^37^
CSN3	Osteosarcoma	Oncogene	Interacts with Beclin1 and Raf-1, subsequently inducing EMT; regulates expression of TP53 and MAPK	^42,43,48^
	Prostate cancer	Oncogene	CSN3 knockdown decreases phosphorylated p38 MAPK levels and impairs EMT	^44^
	HCC	Oncogene	CSN3 knockdown induces growth arrest and apoptosis	^45^
	Lung cancer	Oncogene	Blocks cell cycle progression	^46,47^
	Kidney cancer	Oncogene	Regulates phospho-AKT(Thr308), cyclin D1, and caspase-3 expression	^49^
CSN4	Prostate cancer	Oncogene	Down-regulates p53 expression and up-regulates sGCα1 expression	^55^
	Breast cancer	Oncogene	Alters the proliferation and apoptosis of cancer cells	^56^
Jab1/CSN5	Gastric cancer	Oncogene	Down-regulates p14ARF expression and modulates p53-related apoptotic pathways	^57,58^
	Breast cancer	Oncogene	Up-regulates Rad51 in a p53-dependent manner; affects apoptosis and G1 phase cell cycle arrest in cancer cells	^59,60^
	HCC	Oncogene	Negatively correlated with p57 levels	^61^
	Non-small cell lung cancer	Oncogene	Facilitates cancer cell growth *via* stabilizing survivin	^62^
	Osteosarcoma	Oncogene	Accelerates tumor formation in a p53-dependent manner	^63^
	Prostate cancer	Oncogene	Controls critical oncoproteins	^64^
	Glioma	Oncogene	Regulates the Siah1/β-catenin pathway	^65^
	Laryngeal cancer	Oncogene	Negatively correlated with caspase-3 cleavage and p53 expression	^66^
CSN6	Breast cancer	Oncogene	Positively regulates Snail1 stability; correlated with mutant-type p53 protein	^80,81^
	HCC	Oncogene	Promotes EMT by inhibiting E-cadherin	^82^
	CRC	Oncogene	Positively correlated with ERK2 activation and β-catenin expression	^83^
	Gastric cancer	Oncogene	Ubiquitin-independent proteasomal degradation of p16^INK4a^	^84^
	Pancreatic cancer	Oncogene	Stabilizes c-Fos protein by binding and decreasing its ubiquitination	^85,86^
	Melanoma	Oncogene	Controls UBR5-mediated ubiquitination and degradation of CDK9	^87^
	Glioblastoma	Oncogene	CHIP-mediated degradation of EGFR	^88^
	Oral squamous cell carcinoma	Oncogene	Down-regulates TIMP-2	^89^
CSN7A	Gastric cancer	Tumor suppressor	Promotes deubiquitination of IκBα and inactivates NF-κB signaling	^92^
CSN7B	HCC	Oncogene	Associated with clinical outcomes of patients	^93^
	RCC	Oncogene	CSN7B loss inhibits cancer cell proliferation and invasion	^94^
CSN8	CRC	Oncogene	Regulates hypoxia-induced EMT	^95^
	Cutaneous melanoma	Oncogene	Regulates EMT-associated proteins	^96^
	Gastric cancer	Oncogene	As a target of miR-146a, inhibits GPCR-mediated NF-κB activity	^97^

CSN, COP9 signalosome; HCC, hepatocellular carcinoma; EMT, epithelial-mesenchymal transition; CRC, colorectal cancer; EGFR, epidermal growth factor receptor; TIMP-2, tissue inhibitor of metalloproteinase 2; RCC, renal cell carcinoma; GPCR, G protein-coupled receptor.

### Dual roles of CSN1 in cancer

CSN1 plays a critical role in the ubiquitin-proteasome pathway and regulates various cellular processes, including the cell cycle and DNA repair^29^. Analysis of transcriptomic data from The Cancer Genome Atlas (TCGA) has revealed that CSN1 expression is enhanced in hepatocellular carcinoma (HCC) compared with normal tissue. High levels of CSN1 indicate poor prognosis in patients with HCC. Mechanistically, CSN1 promotes the migration and proliferation of HCC cells by upregulating cyclin A2 expression^30^. However, Feber et al.^31^ have suggested that CSN1 is a novel tumor suppressor that disrupts miRNA-mediated gene silencing in penile squamous cell carcinoma when mutated. In addition, CSN1 has been reported to suppress mitogen-activated signal transduction^32^ and has been implicated in the activation of p53^33^. However, few studies have attempted to explain the functional role of CSN1 in cancer. Therefore, the role of CSN1 in cancer development must be further defined.

### Dual roles of CSN2 in cancer

CSN2 functions as a transcriptional corepressor and facilitates pluripotency maintenance^34^. CSN2 is considered a putative tumor suppressor gene and has diminished expression in tumor tissues^35^. Carvalho et al.^36^ have shown that CSN2 might function as a tumor suppressor in colorectal cancer (CRC), and CSN2 could act as a candidate target gene of miR-15a-3p for preventing colorectal adenoma-to-carcinoma progression. Serum CSN2 levels serve as a prognostic marker in gastric cancer, and low serum CSN2 is associated with poor survival in patients with gastric cancer^37^. Furthermore, patients with low CSN2 expression have a higher risk of metastasis/recurrence. Multivariate Cox analysis has identified CSN2 as an independent prognostic factor for overall survival and disease-free survival in patients with CRC^38^. These data collectively indicate the tumor-suppressive role of CSN2 in human cancers. However, CSN2 has been found to inhibit the degradation of Snail in cancer cells^39^. Silencing of CSN2/Snail diminishes radiation-induced cell migration and epithelial-mesenchymal transition (EMT) of HCC cells^40^. Therefore, clarifying the role of CSN2 in cancer is essential.

### CSN3 as an oncogene in cancer

CSN3 is crucial for the maintenance of cell proliferation in mouse embryonic epiblasts and is associated with cancer progression and metastasis^41^. Knockdown of the CSN3 gene has been found to decrease the metastasis of osteosarcoma cells^42^. In addition, CSN3 knockdown suppresses metastasis of osteosarcoma cells to the lungs, both *in vitro* and *in vivo*^43^. The silencing of CSN3 also impairs EMT in osteosarcoma cells and prostate cancer^43,44^. Furthermore, CSN3 has been reported to be a crucial regulator of cell survival and the cell cycle, and to mediate HCC cell proliferation^45^. CSN3 deletion restrains lung cancer tumor growth by repressing cell cycle progression^46,47^. Moreover, overexpression and amplification of CSN3 in osteosarcomas has been found to disrupt the anti-tumor pathway by targeting TP53 for proteasome-mediated degradation^48^. CSN3 may promote kidney cancer progression by regulating cyclin D1, caspase-3 expression and phospho-AKT (Thr308)^49^. Moreover, the CSN3 gene is located on chromosome 17p11.2˜p12, an unstable chromosomal region that is frequently amplified in osteosarcomas and multiple myelomas^50–54^. Therefore, targeting CSN3 may be a promising strategy for anti-tumor therapy.

### CSN4 overexpression in cancer

Few studies have investigated the role of CSN4 in cancer biology. Bhansali et al.^55^ have demonstrated that CSN4 protein expression is robustly enhanced in prostate cancer. Furthermore, CSN4 promotes prostate cancer cell proliferation *via* down-regulating p53 protein and up-regulating sGCα1. In addition, TCGA database analysis has indicated differences in CSN4 mRNA levels between tumor and normal tissue samples. CSN4 alters apoptosis and proliferation in breast cancer cells and thereby promotes tumorigenesis^56^. Thus, the role and mechanism of CSN4 in human cancer require further exploration.

### Jab1/CSN5 overexpression in cancer

Aberrant overexpression of Jab1/CSN5 has been implicated in many types of human malignancies, including gastric cancer^57,58^, breast cancer^59,60^, HCC^61^, non-small cell lung cancer^62^, osteosarcoma^63^, prostate cancer^64^, glioma^65^, laryngeal cancer^66^ and many others^67^. Yuan et al.^68^ have examined the differential expression of Jab1/CSN5 between various cancer tissues and corresponding normal tissue samples by querying the Oncomine database. Their results indicated overexpression of Jab1/CSN5 mRNA in central nervous system cancer, bladder cancers, myeloma, and breast cancer in 7 of 475 analyses. TCGA cohort and Gene Expression Omnibus dataset analyses have indicated that Jab1/CSN5 expression is significantly enhanced in cervical cancers compared with normal tissue. High Jab1/CSN5 expression is associated with unfavorable clinical outcomes in patients with cervical cancer^69^. These statistical data indicate the role of Jab1/CSN5 as a biomarker and therapeutic target in numerous cancers. Furthermore, Jab1/CSN5 overexpression tends to correlate with cancer cellular proliferation^70^, vascular invasion^71^, lymph node metastasis^72^ and histological differentiation clinical stage^73^. Indeed, Jab1/CSN5 promotes tumorigenesis *via* degrading several substrates, such as p53^74^, p27^75^, p14ARF^57^, Smad4^76^, and the WNT inhibitor DKK1^77^. These targets of Jab1/CSN5 function as tumor suppressors involved in various cellular processes, such as proliferation, apoptosis, angiogenesis, and the cell cycle^78^. Overall, marked progress has been made in verifying the important role of Jab1/CSN5 in tumor development. Jab1/CSN5 may serve as a novel biomarker of poor prognosis in human cancers.

### CSN6 overexpression in cancer

CSN6 is overexpressed in many types of cancer, according to analysis of human cancer patient data sets from Gene Expression Omnibus and Oncomine^79^. Furthermore, studies increasingly demonstrate that CSN6 expression is enhanced in various human cancers, including breast cancer^80,81^, HCC^82^, CRC^83^, gastric cancer^84^, pancreatic cancer^85,86^, melanoma^87^, glioblastoma^88^, and oral squamous cell carcinoma^89^. These studies have indicated that CSN6 is associated with the occurrence and development of carcinogenesis. Furthermore, according to System for Integrative Genomic Microarray Analysis (SIGMA) evaluation, genetic loss or gain of CSN6 (mapped to 7q22.1), and amplification of the CSN6 genomic region are frequently detected in various types of cancer. CSN6 gene copy number is positively correlated with tumor size^90^. Correspondingly, high CSN6 expression is significantly correlated with TNM stage, depth of invasion–pT status and lymph node metastasis–pN status in gastric cancer, breast cancer, and pancreatic adenocarcinoma^80,84,86^. Kaplan-Meier analysis has indicated that higher CSN6 expression is associated with shorter overall survival in patients with pancreatic cancer, HCC, or CRC^82,83,85^. In addition, CSN6 gene mRNA levels, assessed from TCGA data, have revealed higher CSN6 expression in tumor tissue than in normal tissue, and indicated that amplification of the CSN6 gene is associated with advanced disease stage^83^. Collectively, all these data suggest that CSN6 is a potential diagnostic biomarker and interference target for the treatment of human cancer.

### CSN7A or CSN7B paralogs in cancer

CSN exists as 2 variant complexes containing either CSN7A or CSN7B paralogs that have overlapping functions in the deneddylation of CRL^91^. CSN7A mRNA and protein levels, evaluated by qRT-PCR and immunohistochemistry, are significantly lower in gastric cancer tissues than normal gastric tissues. Furthermore, lower CSN7A expression is associated with clinical manifestations, including positive lymph node metastasis and larger tumor size^92^. These data indicate the tumor-suppressive role of CSN7A in gastric cancer. According to data presented in Kaplan–Meier plotter and cBioPortal, CSN7B is associated with increased recurrence rates and decreased survival time in HCC and renal cell carcinoma (RCC)^93,94^. Furthermore, CSN7B deletion inhibits RCC cell invasion and proliferation^94^. Collectively, these studies suggest an oncogenic role of CSN7B in HCC and RCC.

### CSN8 overexpression in cancer

Studies have revealed that CSN8 is a critical regulatory molecule that controls EMT, thus endowing CRC cells with vigorous invasion capability and highly aggressive metastatic characteristics^95^. Furthermore, CSN8 is elevated in cutaneous melanoma, and it accelerates cancer progression *via* regulation of EMT^96^. In addition, Crone et al.^97^ have revealed that CSN8 is a target of microRNA-146a, which inhibits the activation of NF-κB-regulated tumor-promoting cytokines and growth factors in gastric cancer. These data indicate that CSN8 is an oncogene. However, the mechanisms underlying the role of CSN8 must be further explored.

## Transcriptional regulation

The CSN was initially discovered as a transcriptional repressor of light-dependent growth in *Arabidopsis*^14^. This repression was attributed to the function of the CSN in regulating the stability of transcription factors that are usually unstable and degraded in darkness^98^. The CSN is structurally similar to the eukaryotic translation initiation factor eIF3 and is involved in the regulation of eIF3^99^. This section evaluates the role of the CSN as a transcriptional regulator in cancer. The CSN regulates transcription factor stability and transcriptional activity through the removal of the ubiquitin-like modifier NEDD8 from CRLs. For example, CSN promotes the deubiquitination of IκBα, thereby attenuating the activity of the transcription factor nuclear factor κB (NF-κB)^100,101^. Moreover, CSN2 and Jab1/CSN5 act as transcriptional corepressors and coactivators, respectively^102^.

Jab1/CSN5 was initially identified as a transcriptional co-activator of c-Jun, which acts *via* activator protein 1 (AP-1) sites^103^. AP-1 transcription factors are associated with progression and recurrence of various cancers^104,105^. MK2-mediated phosphorylation of Jab1/CSN5 facilitates recruitment of Jun to AP-1 sites, thereby augmenting AP-1 activity in triple-negative breast cancer^106^. Recent studies have indicated the role of Jab1/CSN5 as a regulator of transcription factor activity. The transcription factor NF-κB is a critical regulator of many physiological and pathophysiological processes associated with carcinogenesis, including cell growth, apoptosis, and survival^107^. Jab1/CSN5 silencing may arrest cell cycle progression and inhibit invasion *via* decreasing the transcriptional activity of NF-κB in colorectal cancer^108^. Furthermore, Jab1/CSN5 knockdown regulates signal transducer and activator of transcription 3 (STAT3) DNA-binding activity and increases STAT3 expression *via* protein-protein interaction in colon cancer cells^109^. E2F transcription factors are crucial regulators of cell cycle progression and cell fate control, and are induced by the E2F1 binding partner Jab1/CSN5. Increasing evidence indicates the role of Jab1/CSN5 in a variety of human cancers. Lu et al.^110^ have suggested that the pro-proliferative function of Jab1/CSN5 may be attributable to E2F dependent apoptosis and mitosis. In addition, Jab1/CSN5 promotes the cytoplasmic localization and degradation of the transcription factor RUNX3, which plays crucial roles in fundamental cellular processes associated with tumor development^111^. Furthermore, Jab1/CSN5 promotes pancreatic cancer invasion and metastasis by stabilizing a Fos family protein [Forkhead box M1 (FOXM1)]^112^. The precise mechanism through which Jab1/CSN5 regulates transcriptional activity remains to be investigated. Jun proteins have been suggested to bind Fos proteins to form the gene-regulatory protein AP-1^103^. Jab1/CSN5 or the holo CSN complex interacts with histone methyltransferases and chromatin, thereby regulating transcription^113,114^. The precise mechanism through which Jab1/CSN5 regulates transcriptional activity remains to be investigated.

CSN2, also known as Alien, was initially recognized as a corepressor of steroid hormone signaling^115^. CNS2 directly binds chromatin and enhances NAP-1-mediated nucleosome assembly, thereby contributing to gene silencing^116^. Moreover, CSN2 acts as a corepressor of E2F1 and is involved in cell proliferation^117^. In addition, CSN2 affects transcription factors through ubiquitination. For instance, NF-κB is required for the induction of CSN2, which in turn inhibits the ubiquitination and degradation of the transcription factor Snail^39^. Other subunits, such as CSN6, also attenuate transcription factor expression through ubiquitination in cancer cells^79,80,86^. However, the regulatory mechanisms of the CSN complex and its individual subunits in regulating gene expression remain to be further investigated.

## Cell cycle

**Figure 2:**
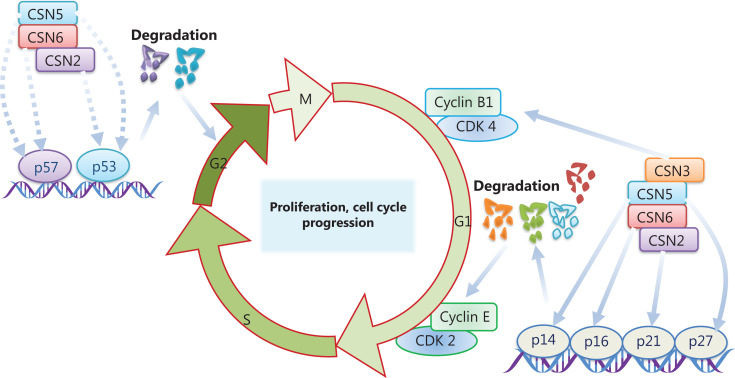
CSN subunits are involved in cell cycle progression. CSN2, CSN3, CSN5, and CSN6 control multiple cell cycle regulators, thereby facilitating cell cycle progression.

Multiple tumor types have been shown to be dependent on CSN-mediated modulation of cell cycle regulators (a finding that could potentially be used in anti-tumor therapies). The CSN and its subunits are involved in the regulation of cell-cycle progression (**Figure 2**). The first link between the CSN and cell cycle progression is that Jab1/CSN5 facilitates degradation of the cyclin-dependent-kinase inhibitor p27 and subsequent G1 phase arrest^118^. As an inhibitor of cyclin E-CDK2, p27 blocks the G1 to S transition in cell cycle progression and is a putative suppressor of tumorigenesis^119–121^. In addition, the cytoplasmic shuttling of p27 is associated with poor survival in patients with cancer^122,123^. The CSN complex and CSN5/CSN6 induce the cytoplasmic translocalization and subsequent degradation of p27^124,125^. Indeed, accumulating evidence indicates that Jab1/CSN5 upregulation is inversely correlated with p27 levels and is associated with poor survival in several human cancers^75,108^. The CSN also plays an essential role in the regulation of other cell cycle-associated gene expression. For instance, Jab1/CSN5 promotes the ubiquitination and degradation of p53 and antagonizes the transcriptional activity of p53^126,127^. CSN6 facilitates cancer cell growth through MDM2-mediated p53 degradation^128,129^. CSN acts upstream of the ubiquitin E3 ligase Skp2, which controls G1/S cell cycle regulators^130^. CSN6 promotes Skp2-mediated degradation of the cyclin-dependent kinase inhibitor p57^131^, whereas Jab1/CSN5 promotes p57 degradation independently of Skp2 in HCC^61^. In addition to the ubiquitin-dependent degradation of proteins, Jab1/CSN5 and CSN6 promote tumorigenesis *via* ubiquitin independent proteasomal degradation of the cell cycle regulators p16^ INK4a^ and p14ARF^57,84^.

Beyond Jab1/CSN5 and CSN6, other CSN subunits are involved in the regulation of cell cycle progression. CSN2 disruption causes cell proliferation deficiency through the accumulation of p53, cyclin E, and the cyclin-dependent kinase inhibitor p21^Cip1/Waf1132^. CSN2 also restrains p27^kip1^ degradation and blocks G1/S phase progression through deneddylation of SCF Cul1^133^. In lung cancer, CSN3 knockdown blocks cell cycle progression at G0/G1 phase by upregulating p21 and downregulating CDK4 and cyclin B1^46^. In summary, the CSN controls multiple cell cycle regulators and steps in cell cycle progression. Therefore, elucidating the CSN subunits and the subcomplexes’ function in cell cycle regulation will be essential.

## Associated signaling pathways of CSN subunits

**Figure 3:**
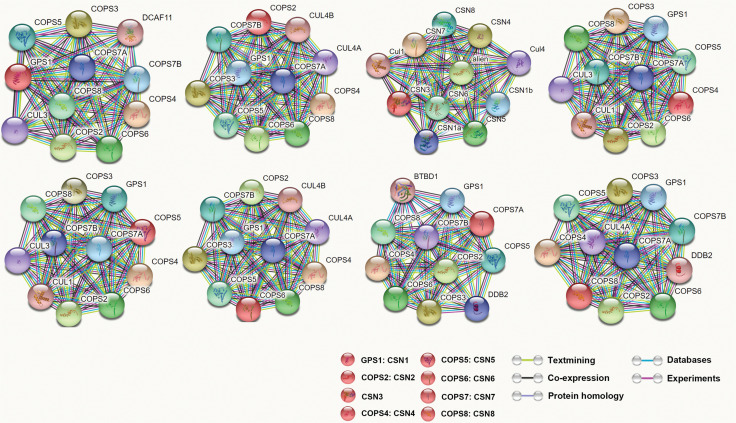
Network of CSN subunit interactors. Evidence view of the STRING database output depicting functional and physical interactors with the CSN subunits proteins (GPS1, COPS2, CSN3, COPS4, COPS5, COPS6, COPS7, and COPS8).

**Figure 4:**
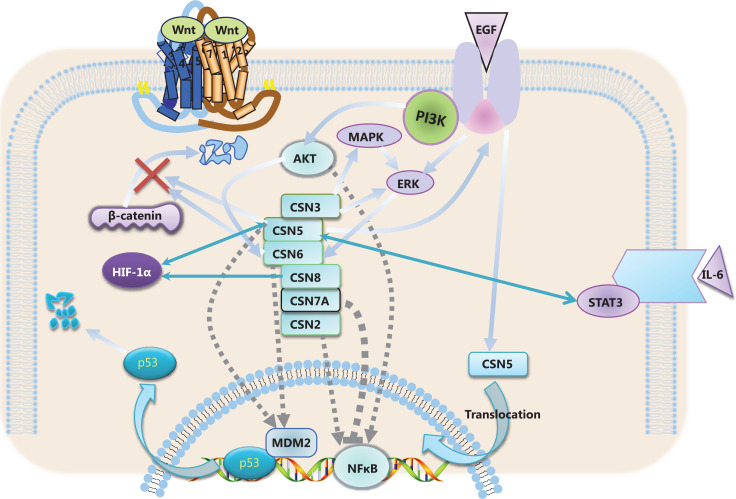
Roles of the CSN subunits in several important signaling pathways.

The CSN lies at the intersection of a range of signaling pathways believed to be critical to tumor development. The network of protein interactions involving CSN is highly complex. The interaction database IntAct (http://www.ebi.ac.uk/intact/) currently lists 7,656 binary interactions for the COP9. To graphically illustrate at least part of this interaction network, we performed a STRING database search (http://stringdb.org/) for proteins interacting either functionally or physically with 8 individual CSN subunits as the input (**Figure 3**). Recent studies have demonstrated that the CSN is involved in several signaling pathways critical to tumor development. Herein, we describe the roles of the CSN in these signaling pathways (**Figure 4**) to improve understanding of tumorigenesis.

### Wnt/β-catenin signaling

The Wnt/β-catenin signaling pathway has crucial roles in angiogenesis and cell proliferation. The CSN regulates Wnt/β-catenin signaling by targeting β-catenin for degradation by the UPS, thus controlling the balance between β-catenin and APC in HeLa cells. Disruption of this balance leads to cancer by promoting cell transformation and metastasis^134^. Wnt/β-catenin signaling is critical to the progression and development of CRC. Jab1/CSN5 depletion affects Wnt signaling by downregulating β-catenin and increases the secretion of the Wnt inhibitor DKK1 in CRC cells^77,135^. In addition, Jab1/CSN5 positively regulates the expression of β-catenin *via* the E3 ubiquitin ligase SIAH-1^136,137^. Thus, these data suggest that Jab1/CSN5 is a promising target for CRC therapy. CSN6 also stabilizes β-catenin expression and facilitates EMT in papillary thyroid cancer cells. Furthermore, CSN6 knockdown sensitizes papillary thyroid cancer cells to FH535 therapy through downregulation of the Wnt/β-catenin signaling pathway^138^.

### PI3K/AKT signaling

PI3K/AKT signaling pathways are frequently perturbed in human cancers and are involved in tumor processes including cell differentiation, growth, and development^139^. A recent study has demonstrated that Jab1/CSN5 enhances epidermal growth factor receptor (EGFR) stability by decreasing EGFR ubiquitination, thereby activating the PI3K/AKT signaling pathway in osteosarcoma cells^140^. Additionally, Jab1/CSN5 silencing may arrest cell cycle progression and inhibit invasion *via* the PI3K/AKT/NF-κB signaling pathway in CRC^108^. Furthermore, macrophage migration inhibitory factor (MIF) is an important inflammatory cytokine involved in tumorigenesis. Lue et al.^141^ have found that Jab1/CSN5 prevents MIF secretion, thereby modulating autocrine MIF-mediated PI3K/AKT signaling in cancer cells. Moreover, PI3K/AKT signaling stimulates the synergistic interaction between Jab1/CSN5 and LASP1, thus promoting CRC progression^72^.

### HER-2 and EGFR signaling

HER-2 plays a critical role in the transformation and growth of cancers in many malignancies, and is associated with poor prognosis in human cancers^142,143^. HER-2 enhances Jab1/CSN5 expression through transcriptional activation in breast cancer^144^. HER-2 transcriptionally increases Jab1/CSN5 promoter activity through the AKT/β-catenin pathway, thus promoting the proliferation of breast cancer cells^145^. In addition, the HER2-AKT signaling pathway positively regulates the stability of CSN6 protein *via* regulating ubiquitin-proteasomal degradation during carcinogenesis^129^. Furthermore, HER-2 encodes a transmembrane tyrosine kinase receptor with extensive homology to EGFR^146^. EGFR-ERK signaling upregulates CSN6 and subsequently enhances programmed cell death ligand-1 (PD-L1) stability in glioblastoma^147^. Moreover, CSN6 positively regulates EGFR stability, thereby promoting glioblastoma proliferation and metastasis^88^. Jab1/CSN5 is also a target of EGFR signaling, and activation of EGFR promotes the translocation of Jab1/CSN5 from the cytoplasm to the nucleus in breast cancer cells^148^. Together, CSN6-associated HER-2 and EGFR signaling may be potential therapeutic targets in cancer.

### ERK and MAPK signaling

The ERK/MAPK signaling pathway is one of the most important pathways involved in cell proliferation. MAPK signaling is downstream of various growth factor receptors, including EGFR^149^. Mechanistic studies have shown that CSN6 is deregulated by EGFR signaling, in which ERK2 interacts with CSN6. Furthermore, high CSN6 levels are positively correlated with ERK2 expression in CRC. Thus, the deregulation of β-catenin by ERK2-activated CSN6 is crucial for CRC development^83^. Moreover, knockdown of CSN3 is associated with downregulation of ERK signaling, thereby decreasing the lung metastasis of osteosarcoma cells^43^. In addition, CSN3 knockdown downregulates the expression of MAPK signaling; therefore, CSN3 may have an essential role in the metastasis of osteosarcoma cells^42^.

### MDM2/p53 signaling

MDM2, a primary cellular inhibitor of p53, is frequently overexpressed in human cancers^150^. CSN-specific phosphorylation targets p53 for ubiquitin-26S proteasome-dependent degradation mediated by MDM2^33^. CSN6 enhances MDM2 stabilization and plays an essential role in promoting tumorigenesis by regulating MDM2/p53 signaling^90^. On the basis of previous studies, we propose that CSN6-mediated stabilization of MDM2 leads to ubiquitination-dependent degradation of p53, thereby interfering with the tumor-suppressive role of p53. Correspondingly, Jab1/CSN5 facilitates MDM2-mediated p53 degradation and promotes p53 nuclear export. Moreover, Jab1/CSN5 overexpression leads to the stabilization of MDM2 and antagonizes the transcriptional activity of p53 in human cancer^127^. Therefore, the CSN is important for modulating p53-mediated tumor suppression by controlling the MDM2/p53 signaling pathway.

### STAT3 signaling

Signal transducer and activator of transcription (STAT) proteins play critical roles in regulating fundamental cellular processes including cell growth and differentiation. Among the STATs, abnormal activation of STAT3 is associated with a variety of human malignancies^151^. Jab1/CSN5 regulates unphosphorylated STAT3 DNA-binding activity *via* protein-protein interaction in colon cancer cells. Likewise, Jab1/CSN5 silencing decreases the expression of STAT3 target genes^109^. Consistently, STAT3 silencing decreases the expression of Jab1/CSN5 and inhibits Jab1/CSN5 promoter activity in cancer cell lines^152^. Furthermore, treatment with the cytokine IL-6 enhances Jab1/CSN5 expression, but this effect is blocked by inhibition of STAT3. These findings indicate that the IL-6/STAT3 signaling pathway is involved in the activation of Jab1/CSN5 transcription^153^. Therefore, the relationship between Jab1/CSN5 and STAT3 associated with carcinogenesis may enhance understanding of the regulatory mechanism of Jab1/CSN5 in human cancer.

### NF-κB signaling

The NF-κB family of transcription factors are key regulators of cell survival and tumor proliferative signaling pathways^154^. NF-κB induced expression of Jab1/CSN5 leads to PD-L1 stabilization and immune suppression in cancer cells^155^. Additionally, NF-κB induced expression of CSN2 blocks the ubiquitination and degradation of the Snail transcription factor, which is required for cell migration and invasion mediated by inflammation^39^. Moreover, activation of NF-κB is necessary for the upregulation of CSN2, which in turn regulates Snail stabilization *via* blocking its interaction with β-TrCP and GSK-3β^156^. In CRC, Jab1/CSN5 knockdown inhibits the secretion of NF-κB and restrains cell proliferation *via* the PI3K/AKT/NF-κB signaling pathway^108^. In gastric cancer, COPS7A promotes IκBα deubiquitination *via* CSN-associated deubiquitinase USP15, then inactivates NF-κB signaling^92^. Thus, CSN-mediated NF-κB signaling may serve as a potential target for the treatment of human cancers in the future.

### HIF-1α signaling

Hypoxia-inducible factor 1 (HIF-1) activates the transcription of genes involved in angiogenesis, cell invasion, and survival. HIF-1α overexpression is associated with elevated patient mortality in many cancer types^157^. CSN increases HIF-1α degradation by promoting the dissociation of HIF-1α from its oxygen-dependent regulator Von Hippel-Lindau (pVHL) in cells^158^. However, Jab1/CSN5 interacts with both HIF-1α and pVHL, thereby stabilizing HIF-1α and positively regulating HIF function^159^. Furthermore, CSN8 partially regulates hypoxia-induced EMT and dormancy by activating the HIF-1α signaling pathway, and it enhances HIF-1α mRNA expression *via* activating NF-κB, thus endowing CRC cells with metastatic and invasive abilities^95^.

## CSN as a therapeutic target

CSN overexpression has been suggested to be generally associated with tumor development in humans, and the development of specific CSN inhibitors is expected to have remarkable effects on cancer treatment. Targeting the CSN may be a productive way to hinder metastasis and proliferation of malignant tumors, and improve chemotherapy and radiotherapy.

The CSN extracted from human erythrocytes has kinase activity and phosphorylates proteins as a consequence of degradation through the ubiquitin pathway. The CSN-associated kinase activity is inhibited by curcumin, emodin, resveratrol, and DRB^160^. Curcumin and emodin also inhibit CSN-associated kinases that trigger proteasome-dependent degradation of Id1 and Id3^161^. Id1 and Id3 are regulators of tumor angiogenesis that are stimulated by CSN-directed c-Jun signaling^162,163^. Among the inhibitors, curcumin is the most studied drug associated with the inhibition of CSN. Curcumin is a yellow plant pigment that induces tumor cell death and apoptosis *via* the inactivation of CSN^164^. Li et al.^165^ have generated a water-soluble polyethylene glycol-conjugated curcumin that inhibits pancreatic cancer cell proliferation by activating Jab1/CSN5. Moreover, this compound sensitizes pancreatic cancer cells to gemcitabine and could potentially be developed as an anti-tumor agent. T83, a novel curcumin analog that induces G2/M arrest and apoptosis, exhibits anticancer activity and induces radiosensitivity through inactivation of Jab1 in nasopharyngeal carcinoma^166^. Another potential target drug is troglitazone, which directly inhibits Jab1/CSN5 promoter activity by repressing Tcf4- and Sp1-mediated transcription. Ectopic expression of Jab1/CSN5 counteracts troglitazone-induced growth inhibition. Animal studies have verified that troglitazone decreases Jab1/CSN5 expression and suppresses HCC cell growth in tumor tissues^167^. These results provide insights into how CSN may be a potential target for anti-tumor therapy. However, drugs targeting the holo CSN complex and other CSN subunits must be further investigated.

Various types of cancers express high levels of PD-L1 and exploit programmed cell death-1 (PD-1)/PD-L1 signaling to evade T cell immunity. Immune checkpoint-blockade treatments targeting PD-1/PD-L1 have consistently shown remarkable anti-tumor effects in patients with advanced cancers^154,168^. Hence, better understanding of the regulatory mechanisms of PD-L1 should provide substantial benefits to patients in cancer diagnosis and immunotherapy. CSN6 and Jab1/CSN5 inhibit the degradation of PD-L1 and subsequently sustain PD-L1 stability in cancer cells^147,155^. In addition, inhibition of Jab1/CSN5 by curcumin decreases the expression of PD-L1 and sensitizes cancer cells to anti-CTLA4 therapy^155^. The anti-inflammatory drug berberine is a negative regulator of PD-L1 and enhanced the sensitivity of tumor cells to co-cultured T-cells. Berberine decreases the expression of PD-L1 and promotes antitumor immunity through inhibiting Jab1/CSN5 activity^169^. Therefore, the relationship between CSN and PD-L1 may enhance understanding of their regulatory mechanism through immune evasion and lead to the development of an efficient cancer therapeutic drug.

CSN is involved in chemotherapy and radiotherapy resistance. CSN2 knockdown suppresses the expression of Snail and enhances the sorafenib sensitivity of HCC cells^170^. Likewise, Jab1/CSN5 silencing reverses the sorafenib resistance of HCC cells and downregulates multi-drug-resistance proteins, including adenosine triphosphate binding cassette (ABC)B1, ABCG2, and ABCC2. Furthermore, repression of Jab1/CSN5 sensitizes cancer cells to cisplatin and ionizing radiation^171^. Together, these findings support that targeting CSN may emerge as a novel therapeutic approach for cancer treatment. Thus, developing effective CSN specific inhibitors for clinical cancer therapy should prove meaningful. In addition, combined treatment with small molecular inhibitors of the associated signaling pathways involving CSN may be a good strategy for the treatment of human cancers.

## Conclusions and perspectives

**Figure 5:**
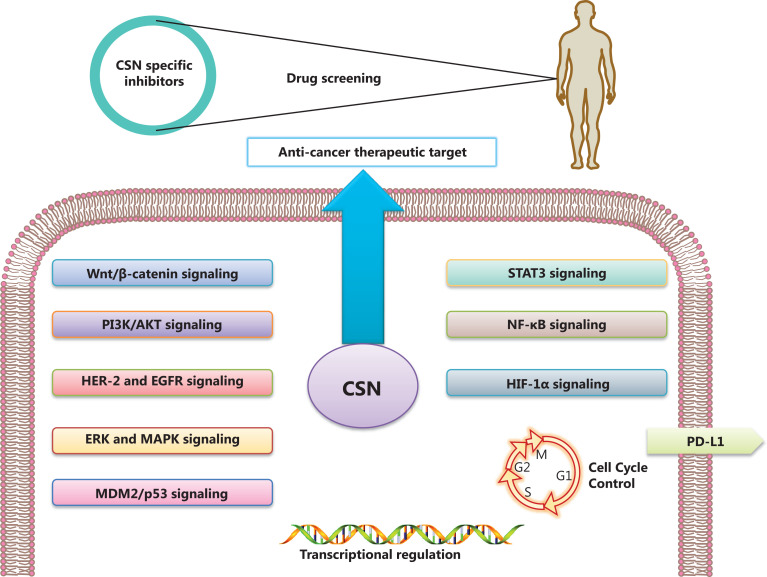
The CSN is involved in a range of cellular processes including transcriptional regulation, cell cycle control, and immune evasion. In addition, the CSN is associated with a complex and diverse network of signaling pathways. A drug screening approach might be required to develop CSN specific inhibitors for cancer therapy.

The CSN, a critical regulator at the intersection between neddylation and ubiquitination, is associated with tumor development. The CSN controls neddylation status of cells by destabilizing the associated DEN1 and through its intrinsic DUB activity. In this review, we discussed the roles of the CSN in cancer, which may be associated with proteasome-mediated protein degradation activity. Recent data (**Figure 5**) have implicated the CSN in a range of cellular processes relevant to cancer progression, such as transcriptional regulation, cell cycle control, and immune evasion. The CSN has multiple prominent functions that affect multiple targets and signaling pathways, which are often carcinogenic. The CSN is activated by a diverse complex network of signaling pathways, which influence one another. Although the CSN subunits have dual roles in tumor development, inhibition of the CSN may be a valuable strategy for the treatment of certain types of cancer.

Current CSN studies based on molecular biology and model organisms support the diverse roles of this complex in cancer. However, a paradox is evident in multiple published studies indicating that the CSN is critical for tumor development. For example, CSN1 and CSN2 have dual roles in cancer progression as oncogenes or tumor suppressors. CSN7B deletion inhibits cell invasion and proliferation, whereas CSN7A is a tumor suppressor. Furthermore, CSN promotes cyclin-dependent kinase inhibition in a manner dependent on or independent of Skp2-mediated degradation in cancer cells. Thus, the detailed mechanistic regulation of the CSN in cancer remains to be investigated. Additional studies are needed to further dissect the function of the CSN and the individual subunits in cancer. Identifying the upstream signals and downstream substrates of the CSN is necessary before efficient therapeutic strategies targeting the CSN can be developed. Crucially, a drug screening approach might be required to develop new inhibitors of the CSN for cancer therapy.
