# Supplementary material for: Targeting the COP9 signalosome for cancer therapy
Source: Cancer Biol Med. 2022 Mar 21;19(5):573–90. doi: 10.20892/j.issn.2095-3941.2021.0605 (PMC9196064; doi:10.20892/j.issn.2095-3941.2021.0605)
Supplement: Supplementary file 1 [file cbm-19-573-s001.pdf]

Supplementary material

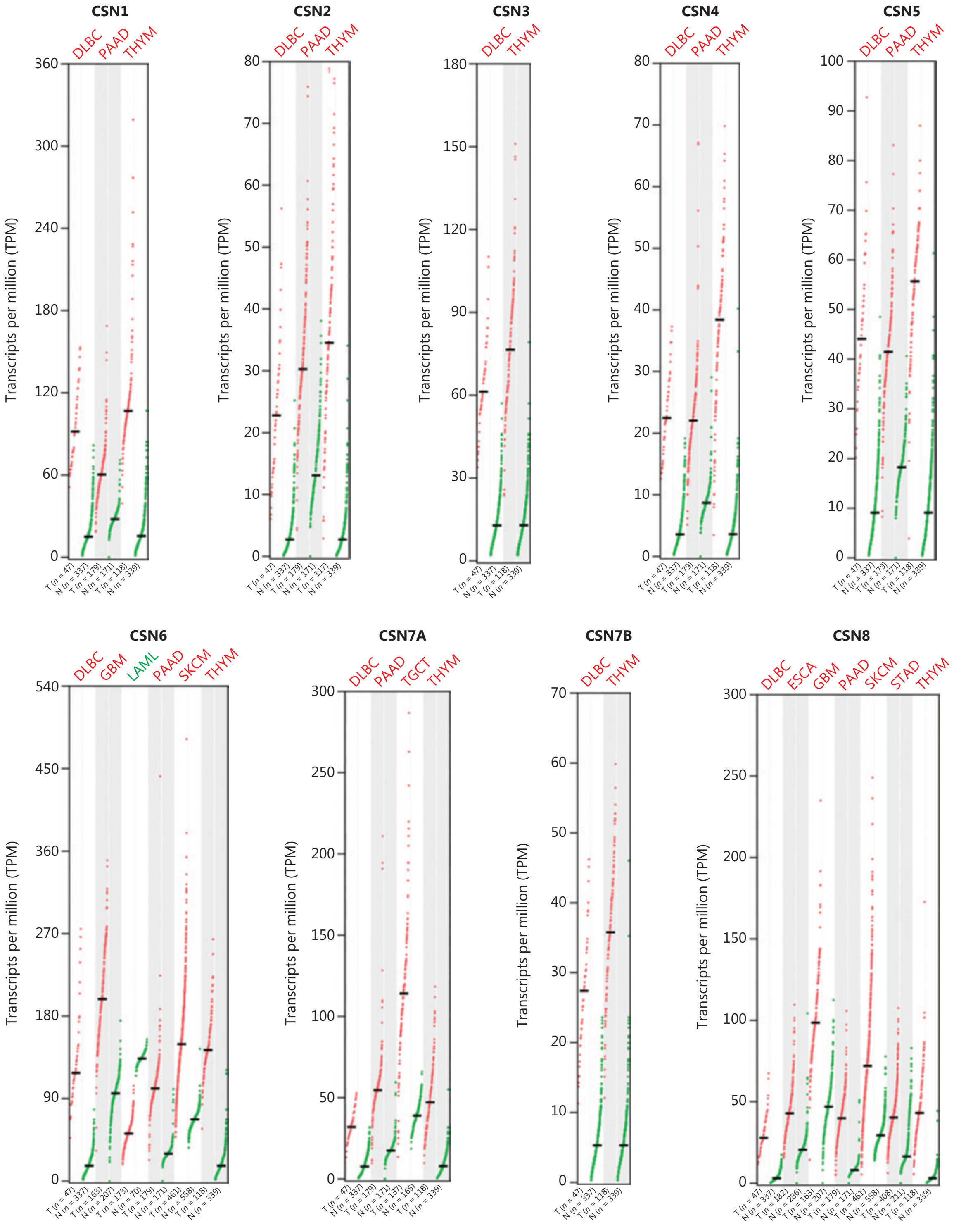

Supplementary Figure S1 Gene expression profiles across tumor samples (T) and paired normal tissues (N) (dot plot). Each dot represents expression levels of samples. Data were obtained from the GEPIA database. DLBC, lymphoid neoplasm diffuse large B-cell lymphoma; PAAD, pancreatic adenocarcinoma; THYM, thymoma; GBM, glioblastoma multiforme; LAML, acute myeloid leukemia; SKCM, skin cutaneous melanoma; TGCT, testicular germ cell tumors; ESCA, esophageal carcinoma; STAD, stomach adenocarcinoma.
